# Evaluation of predictive scores for late and very late recurrence after cryoballoon-based ablation of atrial fibrillation

**DOI:** 10.1007/s10840-020-00778-y

**Published:** 2020-07-07

**Authors:** Makoto Sano, Christian-Hendrik Heeger, Vanessa Sciacca, Niels Große, Ahmad Keelani, Behzad Hassan Hosseiny Fahimi, Huong Lan Phan, Samuel Reincke, Ben Brüggemann, Thomas Fink, Spyridon Liosis, Julia Vogler, Charlotte Eitel, Roland Richard Tilz

**Affiliations:** 1 Medical Clinic II (Department of Cardiology, Angiology and Intensive Care Medicine), University Heart Center Lübeck, University Hospital Schleswig-Holstein, Ratzeburger Allee 160, 23538 Lübeck, Germany; 2 German Centre for Cardiovascular Research (DZHK), Partner Site Hamburg/Kiel/Lübeck, Lübeck, Germany

**Keywords:** Atrial fibrillation, Cryoballoon ablation, Late recurrence, Very late recurrence, MB-LATER score

## Abstract

**Purpose:**

Studies on predictive scores for very late recurrence (VLR) (recurrence later than 12 months) after second-generation cryoballoon-based pulmonary vein isolation (CB2-PVI) are sparse. We aimed to evaluate the frequency of late recurrence (LR) (later than 3 months) and VLR, and to validate predictive scores for LR and VLR after initial CB2-PVI.

**Methods:**

A total of 288 patients undergoing initial CB2-PVI (66 ± 11 years, 46% paroxysmal) were retrospectively enrolled in the LR cohort. In the VLR cohort, 83 patients with recurrence within 3–12 months or with < 12-month follow-up were excluded. The predictive scores of arrhythmia recurrence were assessed, including the APPLE, DR-FLASH, PLAAF, BASE-AF_2_, ATLAS, SCALE-CryoAF, and MB-LATER scores.

**Results:**

During a mean follow-up of 15.3 ± 7.1 months, 188 of 288 (65.2%) patients remained in sinus rhythm without any recurrences. Thirty-two of 205 (15.6%) patients experienced VLR after a mean of 16.6 ± 5.6 months. Comparing the predictive values of these specific scores, the MB-LATER score showed a reliable trend toward greater risk of both LR and VLR (area under the curve in LR; 0.632, 0.637, 0.632, 0.637, 0.604, 0.725, and 0.691 (*p* = ns), VLR; 0.612, 0.636, 0.644, 0.586, 0.541, 0.633, and 0.680 (*p* = 0.038, vs. BASE-AF_2_, respectively)). Kaplan-Meier analysis estimated patients with higher MB-LATER scores which had favorable outcomes (24-month freedom from LR; 26.0% vs. 56.7%, *p* < 0.0001, VLR; 53.4% vs. 82.1%, *p* = 0.013).

**Conclusion:**

The MB-LATER score provided more reliable predictive value for both LR and VLR. Patients with higher MB-LATER scores may benefit from more intensive long-term follow-up.

## Introduction

Pulmonary vein (PV) isolation (PVI) has been positioned as the cornerstone of ablation in patients with atrial fibrillation (AF) [1]. Compared to radiofrequency (RF) ablation, cryoballoon (CB)-based PVI showed non-inferiority concerning clinical outcome and safety in patients with AF [2, 3]. However, recurrence of AF still occurred even in up to 20–30% of paroxysmal (PAF) and 40–60% of non-paroxysmal AF (NPAF) during long-term follow-up [4, 5]. Most AF recurrences are commonly identified within the first year after ablation. In particular, early recurrence within 3 months after the procedure strongly predicted late recurrence (LR) beyond 3 months [6]. On the other hand, very late recurrence (VLR) beyond 1 year, which was observed even in patients with long-term stable sinus rhythm after the initial ablation [7], tends to be overlooked due to sparse follow-up. Hence, a reliable prediction of LR and VLR after catheter ablation of AF is of great clinical importance, serving as a base of an attentive follow-up. Several observational studies reported predictive scores for LR, calculated from clinical characteristics and examinations, such as APPLE [8], DR-FLASH [9], CAAP-AF [10], PLAAF [11, 12], BASE-AF_2_ [13], ATLAS [14], and MB-LATER [15]. Recently published as the novel risk model for VLR after first- and second-generation CB-based PVI, it has been demonstrated that the SCALE-CryoAF score predicted VLR significantly better than the other risk models [16]. However, it remains unclear which is the most reliable predictive score for LR and VLR after initial second-generation cryoballoon-based PVI (CB2-PVI). Further, none of these scores was evaluated specifically for VLR after CB2-PVI. We aimed to evaluate the frequency of LR and VLR and to validate these predictive scores for LR and VLR after initial CB2-PVI in a retrospective patient cohort.

## Methods

### Patient selection

**Fig. 1:**
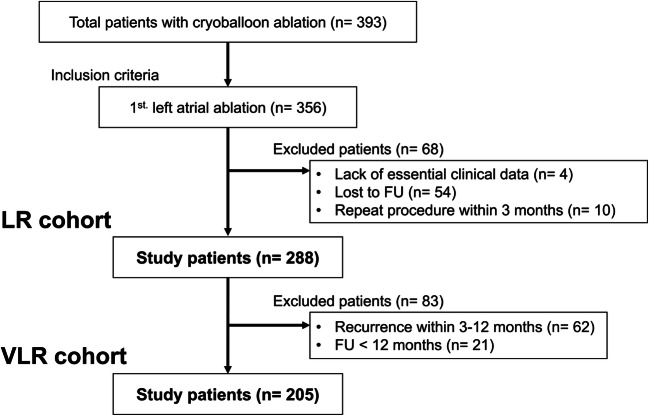
Patient selection. LR, late recurrence; VLR, very late recurrence; FU, follow-up

The present study population included a total of 393 AF patients who underwent CB2-PVI from July 2015 to December 2017 at the University Heart Center Lübeck. Of these patients, 356 patients who underwent initial left atrial ablation were enrolled. Patients enrolled in this study were selected as follows. First, we excluded patients with the following criteria: (1) lost to follow-up within 3 months after the ablation, (2) repeat procedure within 3 months after the ablation; or (3) incomplete clinical data. A total of 288 patients were enrolled in the study cohort (LR cohort). Secondly, focused on VLR, we excluded 83 patients with arrhythmia recurrence 3–12 months after CB2-PVI (*n* = 62) or follow-up less than 12 months (*n* = 21). The VLR cohort consisted of 205 patients (Fig. 1). The recurrence within 3 months after the procedure was judged as early recurrence, 3 months after as LR, and 12 months after as VLR.

AF was defined as paroxysmal if episodes were terminated within 7 days, and as persistent if episodes lasted more than 7 days, including episodes that were terminated by pharmacological or electric cardioversion after 7 days or more [17]. This study was conducted in accordance with the Declaration of Helsinki and was approved by an institutional review committee. Prior to the procedure, all patients provided their informed consent to the procedure and the anonymized analysis of their personal data.

### Preprocedural management

All patients underwent transthoracic and transesophageal echocardiography to measure left ventricular ejection fraction (LVEF), left atrial (LA) diameter, LA area, and LA volume, and to rule out intracardiac thrombi. A blood examination was performed to calculate the estimated glomerular filtration rate (eGFR). Antiarrhythmic drugs (AADs) were continued for the periprocedural period. Anticoagulation therapy was managed as follows: (1) in patients on vitamin-K antagonist, oral anticoagulation therapy was continued aiming at a prothrombin time-international normalized ratio range of 2.0–3.0; (2) in patients under direct oral anticoagulants, one dose was discontinued at the morning of the procedure.

### Intraprocedural management of cryoballoon ablation

The procedure of CB2-PVI has been described in detail before [4]. All procedures were performed under deep sedation with midazolam, fentanyl, and continuous infusion of propofol. Echo-guided vascular access was obtained via the right femoral veins. A ten-pole catheter was inserted into the coronary sinus via the right femoral vein. A single transseptal puncture was performed under fluoroscopic guidance and an 8.5Fr sheath (SL1, Abbott, IL, USA) was exchanged for a 12Fr (Arctic Front Advance, Medtronic, MN, USA) over a wire. The PV ostial anatomy was identified by PV venography. A second-generation 28-mm CB was advanced over an inner lumen mapping catheter (Achieve, Medtronic) into each PV. An optimal PV occlusion was demonstrated by contrast injection. The cryothermal applications lasted at least 180 s for each vein starting with the left PVs, followed by isolation of the right PVs. An additional freeze was delivered only if PV potentials remained. To avoid any esophageal complications, the esophageal temperature was continuously monitored using a temperature probe (SensiTherm, Abbott). For the early detection of a complication with phrenic nerve injury, continuous phrenic nerve stimulation with a maximum output (12 mA, 2.9 ms) and a cycle length of 1000 ms was achieved by a decapolar catheter in the superior vena cava (SVC), measuring the surface compound motor action potential (CMAP) amplitudes and palpating diaphragmatic movement during the freeze of the right PVs. Immediate interruption of the cryothermal application was performed in the case of (1) a transient decline in the diaphragmatic twitching, (2) more than a 30% decrease in the surface CMAP amplitude, or (3) a balloon temperature of less than – 60 °C. In the case of persistent phrenic nerve palsy, no further cryo-application was delivered along the right PVs. Intravenous heparin was administered to maintain an activated clotting time of > 300 s throughout the procedure. In the case of documented typical atrial flutter (AFL), cavotricuspid isthmus (CTI) linear ablation was performed using a 3.5-mm irrigated-tip catheter (Thermocool or Thermocool SF, Biosense Webster Inc., Diamond Bar, CA, USA).

### Post-procedural management and follow-up

Following the procedure, all patients underwent a 24-h Holter electrocardiogram (ECG). On the following day, a 12-lead ECG and a transthoracic echocardiography for exclusion of pericardial effusion were performed. Direct oral anticoagulants were reinitiated 6-h post ablation at half dose, followed by standard dose on the next day. AADs and oral anticoagulants were continuously administered for at least 3 months after the procedure. Regardless of AF types, clinical follow-up with 12-lead ECG and 24-h Holter ECG was regularly carried out after 3, 6, and 12 months (≤ 1 year), every 6–12 months at the outpatient clinic or the referring clinic (> 1 year). In symptomatic cases with suggestive recurrences of AF/atrial tachycardia (AT), a 12-ECG and Holter ECG were added as appropriate. Recurrence of AF/AT was defined as symptomatic or asymptomatic episodes of AF/AT lasting > 30 s.

Repeat procedures were recommended in all patients with symptomatic AF/AT recurrences beyond 3-month blanking period. In patients with repeat procedures, LA-PV reconnection was assessed with LA electroanatomical mapping. In the case of a localized conduction gap between LA and PV, radiofrequency ablation was performed to achieve complete PVI. In patients without LA-PV reconnection, the mapping and ablation strategy targeted non-PV triggers or substrates, including SVC isolation, left atrial appendage (LAA) isolation, LA lines (anterior line, roof line, mitral isthmus line, bottom line, septal line and box isolation, as appropriate), and/or focal ablation, as necessary.

### Score calculation

Based on data from our cohort, we applied the CHADS_2_, CHA_2_DS_2_-VASc, HAS-BLED, and HATCH [18] scores as general scores, and the APPLE [8], DR-FLASH [9], PLAAF [11, 12], BASE-AF_2_ [13], ATLAS [14], and MB-LATER [15] scores as specific scores. The HATCH score consists of hypertension, age ≥ 75 years, transient ischemic attack or stroke (2 points), chronic obstructive pulmonary disease, and heart failure (2 points) with a range from 0 to 7 [18]. The APPLE score involves age > 65 years, persistent AF, impaired eGFR (< 60 ml/min/1.73 m^2^), LA diameter ≥ 43 mm, and LVEF < 50% with a range from 0 to 5 [8]. The DR-FLASH is based on diabetes mellitus, renal dysfunction, persistent AF, LA diameter > 45 mm, age > 65 years, female gender, and hypertension with a range from 0 to 7 [9]. The PLAAF score is composed of persistent AF, LA area, abnormal PV anatomy, AF history, and female gender with a range from 0 to 5 [11]. The BASE-AF_2_ score stands for body mass index > 28 kg/m^2^, LA diameter > 40 mm, smoking, early recurrence within 3 months post-ablation, duration of AF history > 6 years, and NPAF with a range from 0 to 6 [13]. The ATLAS score stands for age > 60 years, female gender (4 points), NPAF (2 points), current smoking (7 points), and indexed LA volume (1 point for each 10 mL/m^2^) with a range from 0 to 15 [14]. The MB-LATER score is calculated by assigning 1 point each for male gender, bundle branch block (i.e., QRS complex duration of ≥ 120 ms), LA diameter ≥ 47 mm and early recurrence within 3 months post-ablation, and 1 or 2 points for persistent or long-standing persistent AF with a range from 0 to 6 [15]. The SCALE-CryoAF score is determined by counting structural heat disease (1 point), coronary artery disease (3 points), LA diameter > 43 mm (1 point), left bundle branch block (3 points), early return of AF (4 points), and NPAF (3 points) with a range from 0 to 15 [16].

For all described scores, we assessed the specific parameters for the initial procedure, followed early recurrence within 3 months post-ablation and calculated these scores.

### Statistical analysis

All categorical variables were presented as number and percentage in each group. Continuous variables were expressed as mean ± standard deviation (SD) or medians (25th, 75th, interquartile range range). Categorical variables were compared between the groups by chi-square or Fisher exact tests, as appropriate. Continuous variables between the groups were examined by an unpaired t test or Mann-Whitney *U* test. The predictive value of tested scores was calculated as the area under the curve (AUC) with 95% confidence interval under the receiver operating characteristic (ROC) curves. The comparison of AUC between each score was evaluated using DeLong test. The optimal cutoff values were determined using Youden’s index. The univariate Cox’s proportional hazards were analyzed separately for each factor of the validated scores. The mean arrhythmia-free survival curves were determined by Kaplan-Meier estimation and compared between the subgroups in the higher or lower MB-LATER score with the cutoff level using the log-rank test. A two-sided *p* value of < 0.05 was considered statistically significant. All statistical analyses were performed using EZR software, which is a graphical user interface for R (The R Foundation for Statistical Computing, Vienna, Austria).

## Results

### Patient characteristics

The LR cohort comprised 288 patients with initial CB2-PVI. The VLR cohort consisted of 205 patients (Fig. 1). The baseline clinical patient characteristics are listed in Table 1. PAF accounted for 133/288 (46%) patients and NPAF for 155/288 (54%) patients. NPAF, hypertension, and bundle branch block were more frequent in patients with LR and VLR. Patients with LR had a significantly lower LVEF and larger LA diameter, LA area, and indexed LA volume. No other significant differences were observed between the groups (Table 1).

**Table 1:** Baseline characteristics

	All patients	LR (+) (*n* = 100)	LR (−) (*n* = 188)	*p* value	VLR (+) (*n* = 32)	VLR (−) (*n* = 173)	*p* value
Gender (male), *n* (%)	183 (63.5)	60 (60.0)	123 (65.4)	0.371	22 (68.8)	112 (64.7)	0.84
Age, years	65.8 ± 10.8	66.2 ± 10.6	65.6 ± 10.9	0.636	65.8 ± 9.8	65.3 ± 10.8	0.806
Age > 65, *n* (%)	169 (58.7)	61 (61.0)	108 (57.4)	0.616	20 (62.5)	99 (57.2)	0.697
BMI	28.6 ± 5.4	28.8 ± 6.5	28.5 ± 4.8	0.624	28.9 ± 7.5	28.6 ± 5.0	0.807
BMI > 28, *n* (%)	135 (46.9)	42 (42.0)	93 (49.5)	0.265	14 (43.8)	86 (49.7)	0.569
Type of AF				<0.001			0.014
Paroxysmal, *n* (%)	133 (46.2)	30 (30.0)	103 (54.8)	<0.001	9 (28.1)	93 (53.8)	0.012
Persistent, *n* (%)	142 (49.3)	63 (63.0)	79 (42.0)	0.001	21 (65.6)	75 (43.4)	0.033
Long-standing, *n* (%)	13 (4.5)	7 (7.0)	6 (3.2)	0.148	2 (6.2)	5 (2.9)	0.3
Duration of AF							
> 3 years, *n* (%)	54 (18.8)	18 (18.0)	36 (19.1)	0.875	6 (18.8)	31 (17.9)	1
> 6 years, *n* (%)	30 (10.4)	12 (12.0)	18 (9.6)	0.547	3 (9.4)	14 (8.1)	0.733
Congestive heart failure, *n* (%)	31 (10.8)	15 (15.0)	16 (8.5)	0.11	2 (6.2)	12 (6.9)	1
Ischemic heart disease, *n* (%)	75 (26.0)	28 (28.0)	47 (25.0)	0.576	7 (21.9)	42 (24.3)	1
Dilated cardiomyopathy, *n* (%)	24 (8.3)	12 (12.0)	12 (6.4)	0.118	4 (12.5)	10 (5.8)	0.241
Hypertrophic cardiomyopathy, *n* (%)	8 (2.8)	4 (4.0)	4 (2.1)	0.455	0 (0.0)	4 (2.3)	1
Hypertension, *n* (%)	229 (79.5)	88 (88.0)	141 (75.0)	0.009	31 (96.9)	129 (74.6)	0.004
Diabetes mellitus, *n* (%)	44 (15.3)	18 (18.0)	26 (13.8)	0.391	5 (15.6)	24 (13.9)	0.784
Vascular disease, *n* (%)	42 (14.6)	17 (17.0)	25 (13.3)	0.483	5 (15.6)	22 (12.7)	0.582
TIA/Stroke, *n* (%)	29 (10.1)	8 (8.0)	21 (11.2)	0.538	1 (3.1)	20 (11.6)	0.21
COPD, *n* (%)	19 (6.6)	7 (7.0)	12 (6.4)	0.809	2 (6.2)	11 (6.4)	1
Current smoking, *n* (%)	45 (15.6)	16 (16.0)	29 (15.4)	1	5 (15.6)	28 (16.2)	1
LVEF, %	52.0 ± 8.7	50.3 ± 9.0	53.0 ± 8.4	0.014	51.3 ± 10.2	53.1 ± 8.3	0.275
LVEF < 50, *n* (%)	77 (26.7)	33 (33.0)	44 (23.4)	0.094	9 (28.1)	41 (23.7)	0.655
LA diameter, mm	42.0 ± 5.6	43.3 ± 5.8	41.4 ± 5.5	0.005	42.4 ± 4.8	41.2 ± 5.3	0.246
LA area, cm^2^	24.6 ± 5.7	26.1 ± 6.6	23.8 ± 5.0	0.001	24.5 ± 4.1	23.6 ± 5.0	0.355
LA area > 21, *n* (%)	206 (71.5)	78 (78.0)	128 (68.1)	0.099	25 (78.1)	114 (65.9)	0.218
LAVI, ml/m^2^	43.4 ± 15.8	47.5 ± 18.4	41.2 ± 13.8	0.001	42.8 ± 11.4	40.4 ± 13.4	0.333
Bundle branch block, *n* (%)	47 (16.4)	27 (27.0)	20 (10.7)	0.001	9 (28.1)	17 (9.9)	0.009
eGFR, ml/min/1.73m^2^	74.7 ± 18.4	74.3 ± 19.4	75.0 ± 17.9	0.751	74.5 ± 18.1	75.2 ± 17.8	0.824
eGFR < 60, *n* (%)	64 (22.2)	26 (26.0)	38 (20.2)	0.298	7 (21.9)	34 (19.7)	0.811

Values are given as the mean ± SD, median (25th, 75th interquartile range), or n (%)

*LR*, late recurrence; *VLR*, very late recurrence; *BMI*, body mass index; *AF*, atrial fibrillation; *TIA*, transient ischemic attack; *COPD*, chronic obstructive pulmonary disease; *LVEF*, left ventricular ejection fraction; *LA*, left atrium; *LAVI* left atrial volume index; *eGFR*, estimated glomerular filtration rate

### Procedural details

Two hundred eighty-six (99.3%) of 288 patients had successful all CB2-PVI. None of the patients needed touch-up ablation using RF energy. Abnormal PV anatomy was recognized as left common PV in 23/288 (8%) patients. The median number of applied freezes were 5 (IQR, 4, 6). More than 2 freezes per PV were delivered in 61/288 (21.2%) of right superior PV (RSPV), 107/288 (37.2%) of right inferior PV (RIPV), 87/288 (30.2%) of left superior PV (LSPV), and 102/288 (35.4%) of left inferior PV (LIPV). CTI ablation was performed in 25/288 (8.7%) patients. Procedure-related cardiac tamponade occurred in 2/288 (0.7%) patients that was managed with percutaneous drainage. Phrenic nerve palsy occurred in 10/288 (3.5%) patients, with 9 cases recovering during follow-up and 1 persisting at 12 months. Puncture-related groin hematoma occurred in 12/288 (4.2%) patients, two of whom required surgical or subcutaneous intervention for complicated aneurysm. Procedural details for patients with and without LR or VLR are summarized in Table 2. The prevalence of abnormal PV anatomy was significantly higher in patients with LR (13/100 [13%] vs 10/188 [5.3%], *p* = 0.037) and VLR (6/32 [18.8%] vs 10/173 [5.8%], *p* = 0.023). Patients with LR had a shorter time to isolation of LIPV (35 [24, 51] vs 44 [28, 61] seconds, *p* = 0.035). No other procedural details showed significant differences in patients with and without LR or VLR.

**Table 2:** Procedural details

	LR (+) (*n* = 100)	LR (−) (*n* = 188)	*p* value	VLR (+) (*n* = 32)	VLR (−) (*n* = 173)	*p* value
Successful PVI, *n* (%)	99 (99.0)	187 (99.5)	1.000	31 (96.9)	172 (99.4)	0.288
Total number of freezes, *n*	5 (4, 6)	5 (4, 6)	0.911	5 (4, 6)	5 (5, 6)	0.951
RSPV						
Number of freezes, *n*	1 (1, 1)	1 (1, 1)	0.411	1 (1, 2)	1 (1, 1)	0.147
Nadir temperature, °C	− 51 (− 56, − 46)	− 50 (− 54, − 46)	0.533	− 48 (− 55, − 46)	− 50 (− 54, − 46)	0.915
Time to isolation, s	29 (21, 48)	30 (20, 40)	0.842	30 (22, 39)	30 (20, 43)	0.664
Duration of freeze, s	180 (180, 240)	180 (180, 240)	0.757	180 (180, 272)	180 (180, 240)	0.680
RIPV						
Number of freezes, *n*	1 (1, 2)	1 (1, 2)	0.862	1 (1, 2)	1 (1, 2)	0.936
Nadir temperature, °C	− 47 (− 51, − 43)	− 47 (− 52, − 43)	0.530	− 46 (− 50, − 43)	− 46 (− 51, − 42)	0.750
Time to isolation, s	35 (24, 65)	46 (26, 80)	0.196	35 (30, 79)	45 (25, 80)	0.977
Duration of freeze, s	240 (180, 360)	240 (180, 360)	0.844	240 (180, 372)	240 (180, 382)	0.911
LSPV						
Number of freezes, *n*	1 (1, 2)	1 (1, 2)	0.588	1 (1, 2)	1 (1, 2)	0.384
Nadir temperature, °C	− 48 (− 53, − 45)	− 48 (− 52, − 45)	0.735	− 49 (− 53, − 43)	− 48 (− 53, − 45)	0.955
Time to isolation, s	34 (25, 47)	32 (27, 46)	0.925	33 (27, 45)	32 (26, 48)	0.767
Duration of freeze, s	240 (180, 360)	240 (180, 358)	0.800	240 (180, 360)	240 (180, 360)	0.494
LIPV						
Number of freezes, *n*	1 (1, 2)	1 (1, 2)	0.899	1 (1, 2)	1 (1, 2)	0.383
Nadir temperature, °C	− 46 (− 51, − 42)	− 45 (− 51, − 41)	0.182	− 46 (− 50, − 43)	− 44 (− 51, − 41)	0.149
Time to isolation, s	35 (24, 51)	44 (28, 61)	0.035	36 (25, 55)	44 (26, 64)	0.345
Duration of freeze, s	240 (180, 360)	240 (180, 360)	0.835	240 (180, 336)	240 (180, 380)	0.845
Contrast volume, ml	115 ± 39	103 ± 32	0.007	110 ± 34	104 ± 32	0.341
Fluoroscopy time, min	19.2 ± 10.2	20.7 ± 12.7	0.346	19.7 ± 10.6	20.8 ± 12.8	0.687
Abnormal PV anatomy, *n* (%)	13 (13.0)	10 (5.3)	0.037	6 (18.8)	10 (5.8)	0.023
CTI linear ablation, *n* (%)	6 (6.0)	19 (10.1)	0.278	1 (3.1)	16 (9.2)	0.482
Acute complication						
Cardiac tamponade, *n* (%)	1 (1.0)	1 (0.5)	1.000	0 (0.0)	2 (1.2)	1.000
Pericardial effusion, *n* (%)	4 (4.0)	2 (1.1)	0.186	0 (0.0)	3 (1.7)	1.000
Phrenic nerve palsy, *n* (%)	4 (4.0)	6 (3.2)	0.741	1 (3.2)	6 (3.5)	1.000
Hematoma, *n* (%)	5 (5.1)	7 (3.8)	0.758	1 (3.2)	7 (4.1)	1.000

Values are given as the mean ± SD, median (25th, 75th interquartile range), or *n* (%)

*LR*, late recurrence; *VLR*, very late recurrence; *PVI*, pulmonary vein isolation; *RSPV*, right superior pulmonary vein; *RIPV*, right inferior pulmonary vein; *LSPV*, left superior pulmonary vein; *LIPV*, left inferior pulmonary vein; *PV*, pulmonary vein; *CTI*, cavotricuspid isthmus;

### Clinical outcome

During a mean follow-up of 15.3 ± 7.1 months, 188 of 288 patients (65.2%) remained in sinus rhythm without any recurrences including 17 patients on AADs. In the 100 patients with recurrent arrhythmia, the median time to recurrence was 6 [4, 12] months. Of these, 6 patients had only early recurrence. Sixty-two patients had recurrence within 3–12 months, and 32 patients experienced VLR after a mean of 16.6 ± 5.6 months. Kaplan-Meier analysis of 24-month AF/AT-free survival demonstrated a preferable outcome in patients with PAF compared to those with NPAF (62.3% [95% confidence intervals; 0.467–0.756] vs 38.1% [0.258–0.503], *p* < 0.001). Regarding the types of recurrent arrhythmias, patients with PAF developed recurrence of PAF in 20/30 (66.7%), persistent AF in 5/30 (16.7%), and AT or AFL in 5/30 (16.7%), while those with NPAF recurred as PAF in 24/70 (34.3%), persistent AF in 28/70 (40%), and AT or AFL in 18/70 (25.7%) of the patients. Of 100 patients with LR, 57/100 patients (57%) underwent repeat ablation 12.0 ± 6.4 months after the index ablation. LA-PV reconnection was observed in 39/57 (68.4%) patients. The median number of reconnected PVs was 1 (IQR 0, 2): RSPV in 18/57 (31.6%), RIPV in 21/57 (36.8%), LSPV in 26/57 (45.6%), and LIPV in 13/57 (22.8%). LA-PV reconnection was not associated with the presence of abnormal PV anatomy (6/39 [15.4%] vs 5/18 [27.8%], *p* = 0.297). Repeat ablation procedures were described as follows: wide circumferential PVI (elimination of gaps and ostial potentials) in 39/57 (68.4%), CTI ablation in 11/57 (19.3%), focal ablation in 17/57 (29.8%), LA linear ablation in 19/57 (33.3%), LAA isolation in 1/57 (1.8%), and SVC isolation in 1/57 (1.8%) of patients.

Concerning late major adverse events, one patient died from intracranial bleeding after 20 months, one from multiple organ failure after 34 months, and one from septic shock due to infective endocarditis after 4 months. Stroke occurred in 5/288 (1.7%) of the patients after a mean of 15.0 ± 10.2 months. No other major adverse events were observed in this population.

### Predictive scores for LR

**Fig. 2:**
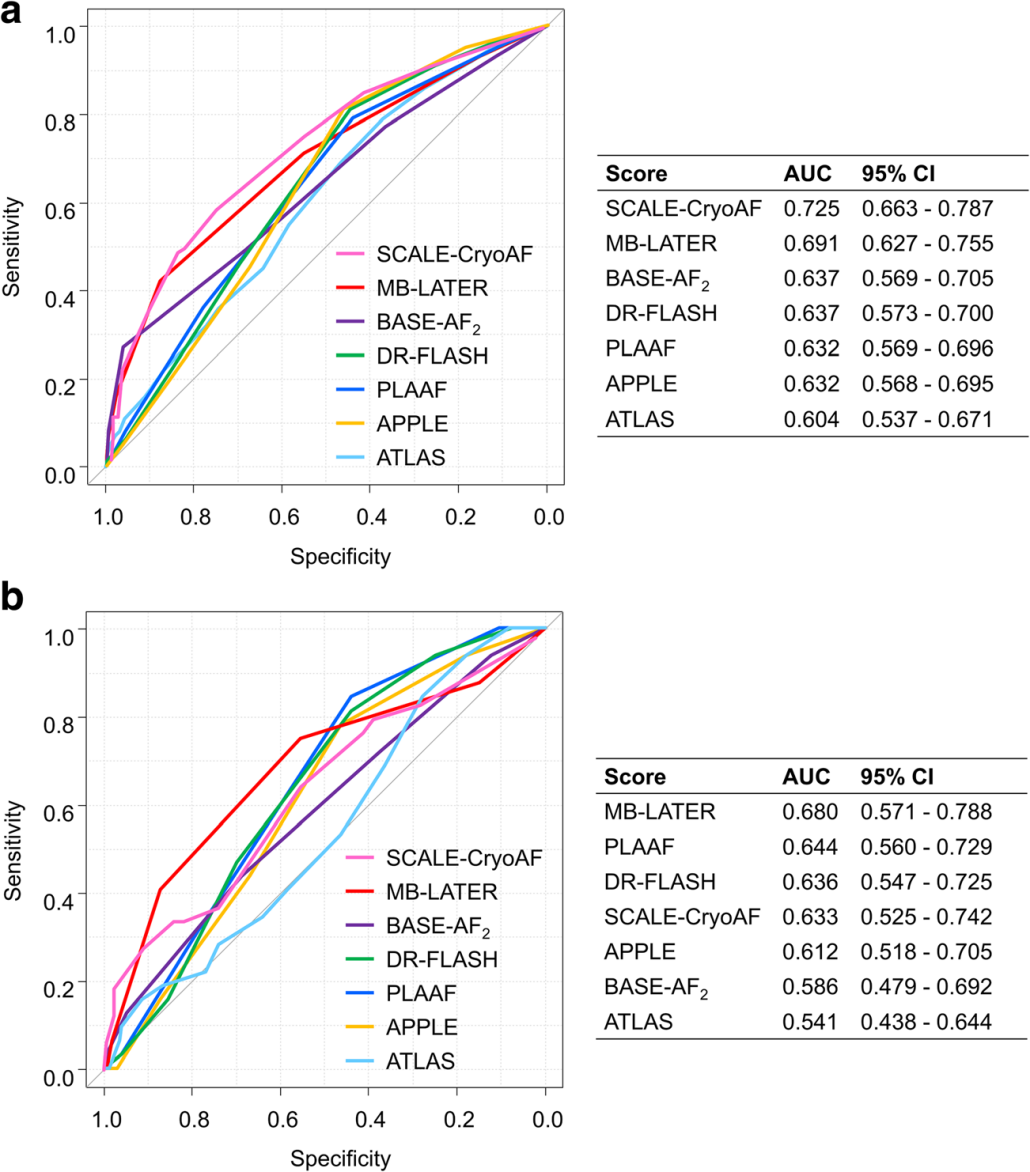
Comparison of specific scores for prediction of LR and VLR. On ROC analysis of specific scores, each score exhibited moderate predictive ability for late recurrence (LR) (**a**) and very late recurrence (VLR) (**b**). Among the specific scores, the MB-LATER score showed the moderate predictive ability for not only LR but also VLR with the high area under curve (AUC). CI, confidence intervals

Predictive scores for LR in this study are shown in Table 3. Regarding general scores, the CHA_2_DS_2_-VASc and HAS-BLED scores were significantly higher in patients with LR than in those without LR, while CHADS_2_ and HATCH score did not differ between patients with or without LR. On ROC curve analysis, all the general scores had negative impacts on prediction of LR (AUC: CHADS_2_ 0.561 [0.497–0.625], CHA_2_DS_2_-VASc 0.570 [0.504–0.637], HAS-BLED 0.588 [0.523–0.653], and HATCH 0.560 [0.496–0.625]). All specific scores, however, were significantly higher in patients with LR than those without LR. The ROC curve analysis of specific scores is illustrated in Fig. 2a. Compared to the AUC among these specific scores by DeLong test, there was no significant difference among these scores in the LR cohort. On univariate Cox’s proportional hazards analysis, the relevance of each factor of the specific scores is listed in Table 4. Regarding the MB-LATER score, bundle branch block (*p* = 0.009), LA diameter > 47 (*p* = 0.01), type of AF (*p* < 0.001), and early recurrence (*p* < 0.0001) were significantly associated with LR. The APPLE score included NPAF (*p* < 0.001) and LA diameter > 43 (*p* = 0.004) as predictors of LR. The DR-FLASH score involved NPAF (*p* < 0.001), LA diameter > 45 (*p* = 0.008), and hypertension (*p* = 0.009) to predict LR. The PLAAF score had only persistent AF (*p* = 0.006) for the prediction of LR. The SCALE-CryoAF score had a positive impact on the prediction of LR in LA diameter > 43 (*p* = 0.009), early recurrence (*p* < 0.0001), and NPAF (*p* < 0.001).

**Table 3:** Predictive scores for LR and VLR

	LR (+) (*n* = 100)	LR (−) (*n* = 188)	*p* value	VLR (+) (*n* = 32)	VLR (−) (*n* = 173)	*p* value
General scores						
CHADS_2_	1.0 (1.0, 2.0)	1.0 (1.0, 2.0)	0.074	1.0 (1.0, 2.0)	1.0 (1.0, 2.0)	0.431
CHA_2_DS_2_-VASc	3.0 (2.0, 4.0)	2.0 (1.0, 4.0)	0.046	2.0 (2.0, 4.0)	2.0 (1.0, 4.0)	0.526
HAS-BLED	2.0 (1.0, 2.0)	1.0 (1.0, 2.0)	0.010	2.0 (1.0, 2.0)	1.0 (1.0, 2.0)	0.105
HATCH	1.0 (1.0, 2.0)	1.0 (1.0, 2.0)	0.075	1.0 (1.0, 2.0)	1.0 (1.0, 2.0)	0.592
Specific scores						
APPLE	2.0 (2.0, 3.0)	2.0 (1.0, 3.0)	< 0.001	2.0 (2.0, 3.0)	2.0 (1.0, 3.0)	0.040
DR-FLASH	3.0 (3.0, 4.0)	3.0 (1.0, 4.0)	< 0.001	3.0 (3.0, 4.0)	3.0 (2.0, 4.0)	0.013
MB-LATER	2.0 (1.0, 3.0)	1.0 (1.0, 2.0)	< 0.001	2.0 (1.8, 3.0)	1.0 (1.0, 2.0)	< 0.001
BASE-AF_2_	2.0 (2.0, 4.0)	2.0 (1.0, 3.0)	< 0.001	2.0 (1.0, 3.0)	2.0 (1.0, 3.0)	0.112
PLAAF	2.0 (2.0, 3.0)	2.0 (1.0, 2.0)	< 0.001	2.0 (2.0, 3.0)	2.0 (1.0, 2.0)	0.007
ATLAS	9.0 (7.0, 12.0)	8.0 (5.0, 11.0)	0.004	8.0 (6.0, 11.0)	8.0 (5.0, 11.0)	0.457
SCALE-CryoAF	5.0 (3.8, 8.0)	3.0 (0, 4.0)	< 0.001	4.0 (3.0, 8.0)	3.0 (0, 5.0)	0.015

Values are given as median (25th, 75th interquartile range)

**Table 4:** Relevance of each factors of predictive specific scores for LR and VLR

	LR (+)		VLR (+)	
	Univariate analysis Hazard ratio (95% CI)	*p* value	Univariate analysis Hazard ratio (95% CI)	*p* value
APPLE				
Age > 65	1.08 (0.72–1.62)	0.710	1.12 (0.54–2.31)	0.770
Non-PAF	2.08 (1.35–3.19)	< 0.001	2.11 (0.97–4.6)	0.061
eGFR < 60	1.28 (0.82–2)	0.280	1.05 (0.45–2.45)	0.900
LAD > 43	1.79 (1.21–2.65)	0.004	1.46 (0.73–2.92)	0.290
LVEF < 50	1.38 (0.91–2.09)	0.130	0.98 (0.45–2.13)	0.970
DR-FLASH				
Diabetes mellitus	1.33 (0.8–2.22)	0.270	1.08 (0.42–2.82)	0.870
eGFR <60	1.28 (0.82–2)	0.280	1.05 (0.45–2.45)	0.900
Non-PAF	2.08 (1.35–3.19)	< 0.001	2.11 (0.97–4.6)	0.061
LAD >45	1.71 (1.15–2.55)	0.008	1.62 (0.8–3.29)	0.180
Age > 65	1.08 (0.72–1.62)	0.710	1.12 (0.54–2.31)	0.770
Gender (female)	1.24 (0.83–1.85)	0.290	0.83 (0.39–1.77)	0.640
Hypertension	2.25 (1.23–4.12)	0.009	11.09 (1.51–81.58)	0.018
MB-LATER				
Gender (male)	0.81 (0.54–1.2)	0.290	1.20 (0.57–2.54)	0.640
BBB	1.81 (1.16–2.82)	0.009	1.58 (0.72–3.45)	0.250
LAD > 47	1.78 (1.15–2.76)	0.010	1.37 (0.56–3.34)	0.490
Type of AF	1.82 (1.3–2.54)	< 0.001	1.87 (1.01–3.47)	0.045
Early recurrence	8.84 (5.79–13.51)	< 0.0001	2.99 (1.14–7.9)	0.027
BASE-AF_2_				
BMI > 28	0.81 (0.54–1.2)	0.290	0.87 (0.43–1.76)	0.710
LAD > 40	1.58 (1–2.49)	0.048	1.12 (0.53–2.4)	0.770
Current smoking	1.15 (0.67–1.96)	0.610	1.18 (0.45–3.08)	0.730
Early recurrence	8.84 (5.79–13.51)	< 0.0001	2.99 (1.14–7.9)	0.027
> 6 years	1.40 (0.76–2.56)	0.280	1.44 (0.43–4.76)	0.550
Non-PAF	2.08 (1.35–3.19)	< 0.001	2.11 (0.97–4.6)	0.061
PLAAF				
Persistent AF	1.77 (1.18–2.66)	0.006	1.79 (0.85–3.77)	0.120
LA area > 21	1.51 (0.94–2.42)	0.091	1.45 (0.62–3.38)	0.390
Abnormal PV anatomy	1.63 (0.91–2.92)	0.100	2.35 (0.96–5.73)	0.061
> 3 years	1.04 (0.62–1.73)	0.890	1.32 (0.54–3.25)	0.540
Gender (female)	1.24 (0.83–1.85)	0.290	0.83 (0.39–1.77)	0.640
ATLAS				
Age > 60	1.18 (0.75–1.85)	0.480	1.07 (0.49–2.33)	0.860
Non-PAF	2.08 (1.35–3.19)	< 0.001	2.11 (0.97–4.6)	0.061
LAVI	1.02 (1.01–1.03)	< 0.0001	1.01 (0.98–1.03)	0.590
Gender (female)	1.24 (0.83–1.85)	0.290	0.83 (0.39–1.77)	0.640
Current smoking	1.15 (0.67–1.96)	0.610	1.18 (0.45–3.08)	0.730
SCALE-CryoAF				
SHD	1.61 (0.98–2.66)	0.063	1.54 (0.72–3.32)	0.268
CAD	1.17 (0.68–2.02)	0.581	0.87 (0.35–2.16)	0.770
LAD > 43 mm	1.93 (1.18–3.15)	0.009	1.47 (0.69–3.13)	0.323
Left BBB	1.73 (0.65–4.63)	0.276	3.82 (1.16–12.5)	0.027
Early recurrence	8.84 (5.79–13.51)	< 0.0001	2.99 (1.14–7.9)	0.027
Non-PAF	2.08 (1.35–3.19)	< 0.001	2.11 (0.97–4.6)	0.061

*CI*, confidence interval; *LR*, late recurrence; *VLR*, very late recurrence; *PAF*, paroxysmal atrial fibrillation; *BBB*, bundle branch block; *eGFR*, estimated glomerular filtration rate; *LAD*, left atrial diameter; *LVEF*, left ventricular ejection fraction; *AF*, atrial fibrillation; *BMI*, body mass index; *LA*, left atrium; *PV*, pulmonary vein; *LAVI* left atrial volume index; *SHD*, structural heart disease; *CAD*, coronary heart disease

### Predictive scores for VLR

Predictive scores for VLR in this study are also listed in Table 3. In patients with VLR, none of the general scores showed a significant difference between the groups. On ROC curve analysis, all the general scores had negative impacts on prediction of VLR (AUC: CHADS_2_ 0.542 [0.458–0.626], CHA_2_DS_2_-VASc 0.535 [0.441–0.629], HAS-BLED 0.586 [0.489–0.684], and HATCH 0.528 [0.445–0.611]). Of the specific scores, the APPLE, DR-FLASH, PLAAF, SCALE-CryoAF, and MB-LATER scores presented higher values in patients with VLR than in those without VLR. The ROC curve analysis of specific scores is demonstrated in Fig. 2b. In the comparison of the AUC among these specific scores by DeLong test, there were significant differences between the MB-LATER and BASE-AF_2_ (*p* = 0.038), and between the PLAAF and ATLAS score (*p* = 0.036) in the VLR cohort. No significant difference was observed between the MB-LATER and the SCALE-CryoAF score (*p* = 0.229) or the PLAAF score (*p* = 0.622). On univariate Cox’s proportional hazards analysis, the influence of each factor of the specific scores is listed in Table 4. Regarding the MB-LATER score, type of AF (*p* = 0.045) and early recurrence (*p* = 0.027) predict VLR significantly. Both the APPLE and PLAAF scores involved no significant factor of the prediction for VLR. The DR-FLASH score involved only hypertension (*p* = 0.018) to predict VLR. The SCALE-CryoAF score included left bundle branch block (*p* = 0.027) and early recurrence (*p* = 0.027) in the positive predictor for VLR.

### MB-LATER score for LR and VLR

**Fig. 3:**
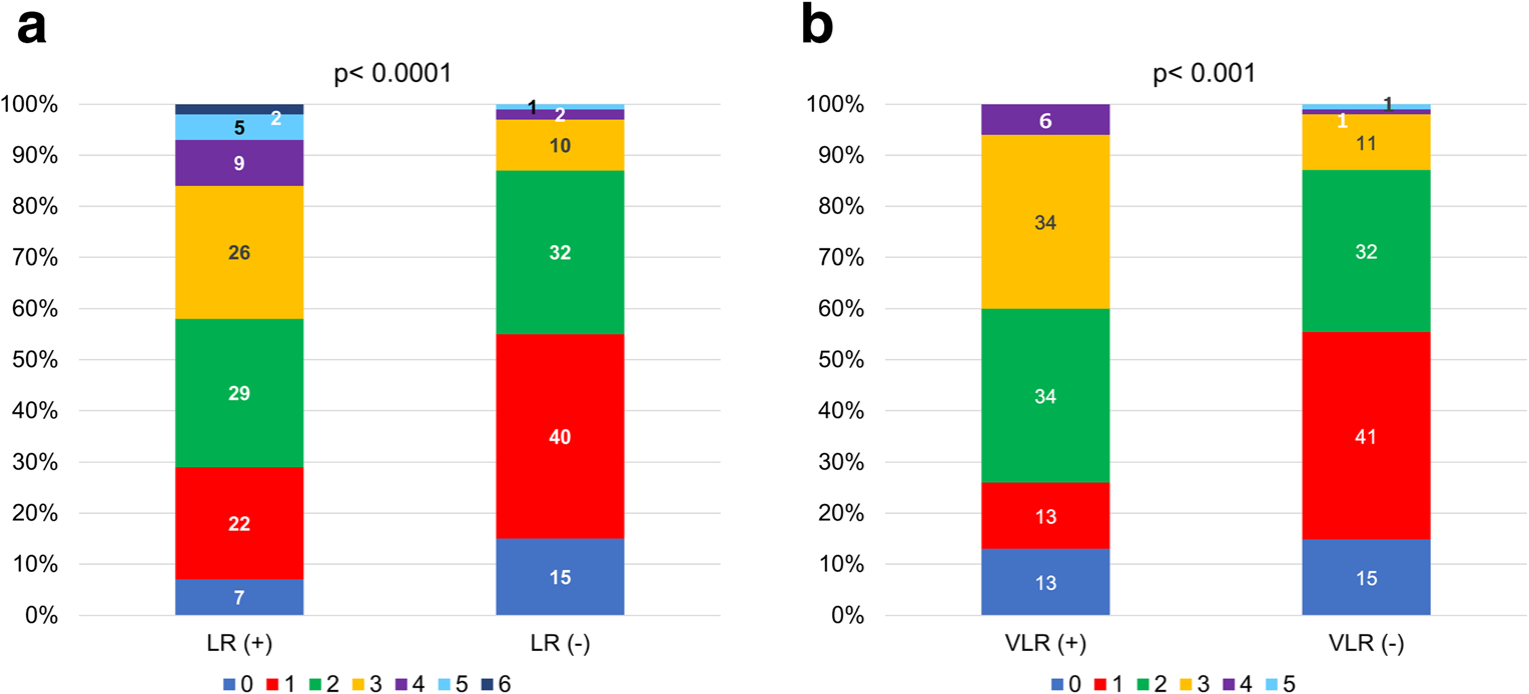
Distribution of the MB-LATER score with and without LR or VLR. The higher MB-LATER score is distributed in both patients with late recurrence (LR) (**a**) and very late recurrence (VLR) (**b**)

Among the specific scores, the MB-LATER score showed the moderate predictive ability for not only LR but also VLR. The MB-LATER score distributed as follows: 0 in 36/288 (12.5%), 1 in 97/288 (33.7%), 2 in 90/288 (31.3%), 3 in 45/288 (15.6%), 4 in 12/288 (4.2%), 5 in 6/288 (2.1%), and 6 in 2/288 (0.7%) of the patients. Distribution of the higher MB-LATER score in both patients with LR and VLR is shown in Fig. 3.

**Fig. 4:**
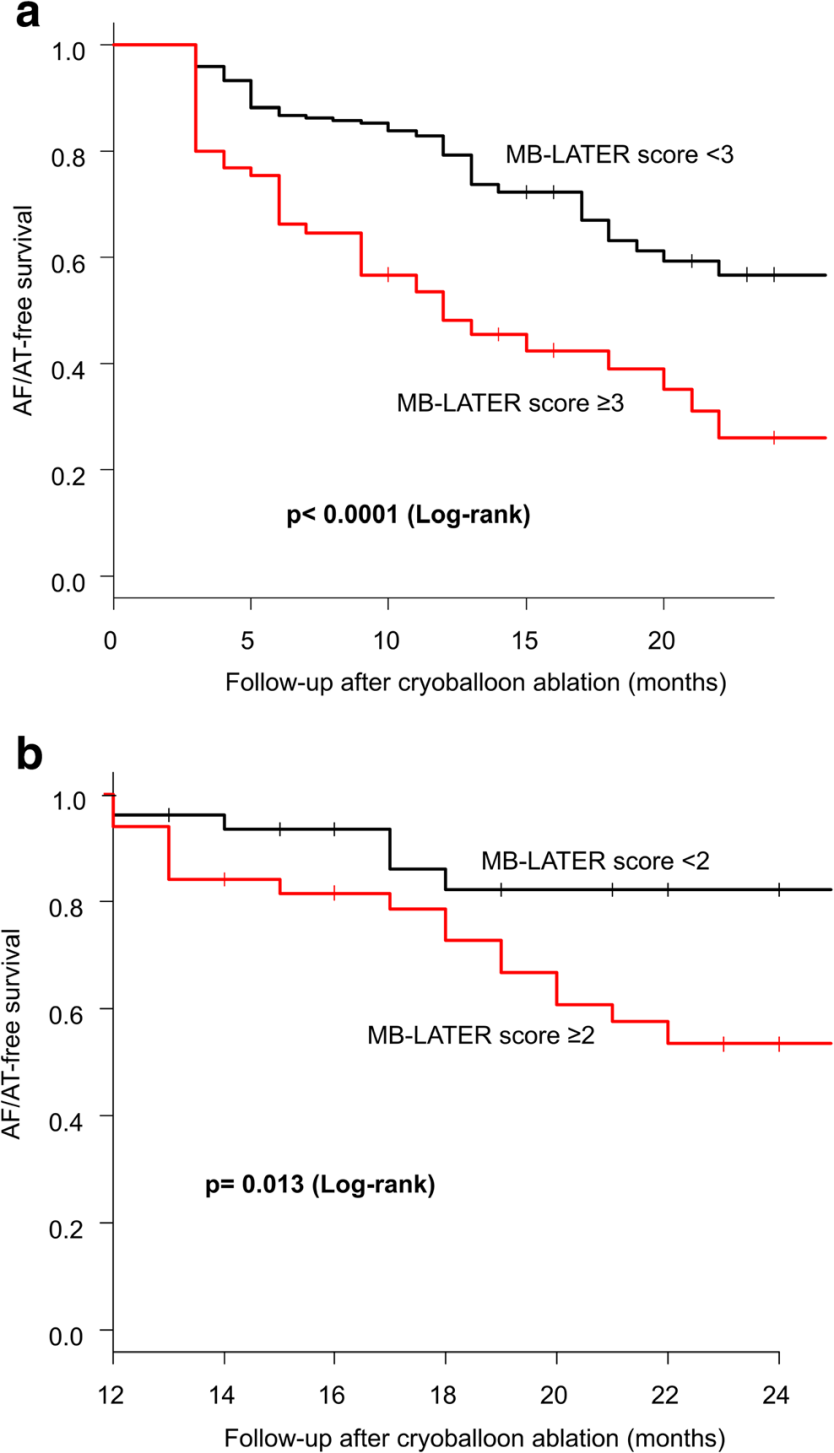
Kaplan-Meier AF/AT-free survival curve according to the MB-LATER score. The cutoff value of the MB-LATER score was **a** ≥ 3 for prediction of late recurrence (LR) and **b** ≥ 2 for very late recurrence (VLR). Kaplan-Meier 24-month AF/AT-free survival presented poor outcome in patients with the MB-LATER ≥ 3 in the LR cohort and ≥ 2 in the VLR cohort (LR; 26.0% vs. 56.7%, *p* < 0.0001, VLR; 53.4% vs. 82.1%, *p* = 0.013)

A sensitivity analysis revealed the optimal cutoff score of ≥ 3 with a sensitivity of 42.0% and a specificity of 87.8% for prediction of LR, and ≥ 2 with a sensitivity of 75.0% and a specificity of 55.5% for VLR. Kaplan-Meier 24-month AF/AT-free survival presented poor outcome in patients with the MB-LATER ≥ 3 in the LR cohort and ≥ 2 in the VLR cohort (LR; 26.0% vs. 56.7%, *p* < 0.0001, VLR; 53.4% vs. 82.1%, *p* = 0.013) (Fig. 4a, b).

## Discussion

The present study focused on the evaluation of several predictive scores for LR and also VLR after initial CB2-PVI. We found that (1) during a mean follow-up of 15.3 ± 7.1 months, 188 of 288 patients (65.2%) remained in sinus rhythm without any recurrences. VLR occurred in 32 of 205 (15.6%) patients; (2) LA-PV reconnection was observed in 39/57 (68.4%) patients who underwent repeat ablations; (3) several specific scores (APPLE, DR-FLASH, PLAAF, SCALE-CryoAF, and MB-LATER) provided a more predictive value for both LR and VLR, in comparison to general scores; (4) the MB-LATER score, which included early recurrence, showed the moderate predictive for both LR and VLR, and so that a high MB-LATER score is a useful tool for identification of patients with a need of intensified follow-up.

### Characteristics of recurrence after CB ablation

Long-term outcome data of more than 3 years after CB2-PVI have reported AF recurrence rate in 20–30% of patients with paroxysmal AF (PAF) and 40–60% of patients with non-paroxysmal AF (NPAF) [4, 5]. In both studies, the outcome of PAF patients was superior as compared to NPAF patients. As recently demonstrated, Akkaya et al. published that a single CB2-PVI retained a favorable 5-year long-term outcome irrespective of types of AF (PAF 61%, NPAF 52%) [19]. The present study had a similar proportion of AF/AT recurrence compared to the previous studies, although more than half of the patients had NPAF and initial LA intervention consisted of a single PVI strategy. Regarding the time point of recurrence, patients with PAF mainly experienced arrhythmia recurrences within 12 months after the procedure, while patients with NPAF developed arrhythmia recurrences irrespective before and after 12 months. In other words, VLR beyond 12 months tended to occur in patients with NPAF. This trend is in line with previous reports [5].

Procedural predictors of late LA-PV reconnection after cryoballoon ablation have been described before [20, 21]. Ghosh et al. revealed that a shorter balloon warming time was the strongest predictor of LA-PV reconnection [20]. Ciconte et al. elucidated that more than 60-s time to isolation and lack of temperatures of − 40 °C within 60-s predicted durable PVI [21]. In our observation, procedural predictors were not found. Generally, durable PVI plays a major role for preventing AF recurrences. CB2-PVI maintained a higher proportion of PVI durability, compared to RF ablation [22, 23]. Conversely, the incidence of LA-PV reconnection after CB2-PVI for PAF was similar between patients with and those without clinical recurrences [24]. Therefore, the mechanism of recurrence after CB2-PVI in patients with PAF differs from RF-PVI, suggesting that non-PV foci and LA substrates may play a role in case of recurrent arrhythmias [25].

### Comparison among predictive scores

Among specific scores for prediction of recurrence, only the BASE-AF_2_, PLAAF, and SCALE-CryoAF scores were derived from CB ablation cohorts [11–13, 16]. The BASE-AF_2_ score includes early recurrence, which greatly contributed to predict AF recurrence (hazard ratio 4.88) in the original cohort [13], similar [15] or superior [26] to the MB-LATER score. D’Ascenzo et al. also mentioned that one of the most powerful predictors of AF ablation failure was early recurrence (odds ratio 4.30) on a meta-analysis [27]. In this study cohort, early recurrence was also the strongest predictive factor of LR (hazard ratio 8.84) and VLR (hazard ratio 2.99), while the other factors of the BASE-AF_2_ score were less reliable markers except NPAF. On the other hand, the PLAAF score is featured by the factor of abnormal PV anatomy [11]. Abnormal PV anatomy may prevent appropriate balloon occlusion, while acute PVI was achieved without increase in complications in previous studies [28, 29]. In our study, there was no relationship between abnormal PV anatomy and LA-PV reconnection in patients with repeat ablation, in spite of more abnormal PV anatomy in patients with LR or VLR.

The RF cohort-derived CAAP-AF score [10], which includes a number of failed AADs as a predictor of recurrence, was verified in a CB cohort, suggesting a score value of ≥ 5 with modest sensitivity of 64% and specificity of 68% [30]. Although the number of failed AADs was a marker for drug refractoriness or severity of AF, our cohort data did not include the number of previous AADs, so we had to exclude the CAAP-AF score from the analysis. In addition, we think that failed AADs cannot always reflect AF severity, for early intervention could be a choice of first-line therapy in the contemporary setting, which contributes to reduce AF recurrence and might prevent progression from paroxysmal to persistent AF [31].

The SCALE-CryoAF score, which was published as the novel risk model for VLR, was superior to other risk models for AF recurrence [16]. The study arose from the first- and second-generation CB-based PVI, while our study focused on the CB2-PVI. In the present evaluation, no significant difference was observed between the MB-LATER and the SCALE-CryoAF score for the prediction of LR and VLR.

In our cohort, LVEF and LA dilatation were related to LR. Besides, NPAF, hypertension, and bundle branch block were associated with both LR and VLR. Collectively, the MB-LATER score, which involved bundle branch block, LA diameter, type of AF, and early recurrence, was available for the prediction of AF recurrence after initial CB2-PVI, suggesting a more predictable tool than CB-derived scores such as the BASE-AF_2_ and PLAAF score.

### The impact of MB-LATER score for LR and VLR

The MB-LATER score, which originated from RF ablation cohorts [15, 32], was published as a predictive marker of VLR which was defined as recurrence following 12-month stable sinus rhythm after ablation. The authors showed that a score of ≥ 2 had the best predictive value for VLR with 75% sensitivity and 73% specificity [15]. Another study validated the predictive ability of the MB-LATER score, highlighting a score of > 2 with modest sensitivity (43%) and specificity (74%) for LR [32]. As recently published, some clinical scores including the MB-LATER score were useful to predict low-voltage areas in the LA [6]. The high MB-LATER score may suggest that a mechanism of recurrence is associated with non-PV foci or LA substrate. The patients with high MB-LATER score may need to maintain AADs for a longer duration and to perform the following ablation target to the substrates, while those with low score may be candidates for early cessation of anticoagulation therapy. Thus, the score-based risk stratification can help to identify patients with a need of longer rhythm follow-up and post-ablative therapy, and to assume the mechanism of recurrence that is useful to consider post-ablative medication of AADs or to select the following ablation strategy.

### Limitation

First, the present study is a single-center study with a retrospective observational design and with only a small number of patients having long-term follow-up. Second, the follow-up was performed by 12-lead ECGs and regular Holter ECGs and therefore asymptomatic episodes might have been missed. Third, we cannot calculate the MB-LATER score at the time point of the procedure, as it is necessary to evaluate early recurrence within 3 months post-ablation. Yet, early recurrence is supported as the major predictor of LR not only from our finding but also from previous studies [6]. Therefore, a score including early recurrence, which can affect the subsequent follow-up strategy, is a preferable and reasonable choice. Fourth, in some patients, recurrences occurred on postprocedural AADs that may influence the rhythm outcome. Fifth, the ROC analysis showed the low AUCs of these scores, suggesting that other potential factors may be associated with the recurrence. Finally, there is a need for further studies to verify our findings in a larger population with longer follow-up or in a prospective design in the future.

## Conclusion

Risk stratification with specific scoring systems can lead to more attentive follow-up strategies. Among several predictive scores, the MB-LATER score provided a more reliable predictive value for both LR and VLR. The patients with a high MB-LATER score may benefit from more intensive long-term follow-up.
